# Arts-based therapies, practices, and interventions in health

**DOI:** 10.1186/s12906-023-04177-4

**Published:** 2023-10-04

**Authors:** Theresa Van Lith, Mark Ettenberger

**Affiliations:** 1 Department of Psychology, Counselling and Therapy, School of Psychology and Public Health, La Trobe University, Melbourne, Australia; 2 Music Therapy Service Clínica Colsanitas, Bogotá, Colombia

## Abstract

This collection focuses on creative art therapies, practices, and interventions in health contexts as part of the Mind-body interventions series of *BMC Complementary Medicine and Therapies.* The collection highlights the emerging value of the arts in complementary medicine and contributes to the expanding knowledge and integration of mind-body interventions and creative art practices.

## Introduction

Creative arts therapies have emerged as a valuable and effective approach within the realm of complementary medicine. These therapies encompass a range of disciplines, including music therapy, art therapy, dance/movement therapy, drama therapy, and writing therapy. They integrate expressive arts practices with psychotherapeutic principles to promote holistic well-being and support the healing process. In recent years, there has been a significant increase in interest and recognition of creative arts therapies within the field of complementary medicine. Research and clinical evidence have demonstrated the profound impacts of these therapies on individuals’ physical, emotional, cognitive, and social wellness, as well as their underlying mechanisms of change [1–3]. Creative arts therapies have been successfully utilized across the lifespan and the wide spectrum from clinic to community, including hospitals, mental health facilities, rehabilitation centers, schools, and community programs, among others.

One of the key strengths of creative arts therapies is their ability to engage individuals on multiple levels. Through artistic expression, individuals are empowered to explore and communicate their thoughts, emotions, and experiences, even when traditional verbal communication may be challenging. In this way, the creative process becomes a transformative tool, facilitating self-discovery, emotional healing, and personal growth. Moreover, creative arts therapies recognize the uniqueness and individuality of each person and take into account both the personal and social/cultural backgrounds of participants, fostering in this way the healthcare institutions’ humanization and person-centered care efforts [4].

Therapists also work collaboratively with clients, tailoring interventions to their specific needs, goals, and resources, promoting a sense of agency, self-empowerment, and self-determination, and allowing individuals to actively participate in their healing journey. Creative arts therapies also provide a non-threatening and non-judgmental space for individuals to express themselves freely [5]. They offer a safe and supportive environment where individuals can explore their inner world, develop coping strategies, enhance self-awareness, and build resilience [6].

By engaging in creative processes supported by a therapeutic relationship, individuals and groups can gain insights, process traumatic experiences, and develop healthier ways of coping with stress, anxiety, and emotional challenges. Furthermore, the integration of creative arts therapies with other complementary medicine modalities has shown promising results. Combining techniques such as mindfulness, meditation, yoga, and energy healing with creative arts practices amplifies the therapeutic benefits, promoting a deeper mind-body connection and facilitating holistic healing [7, 8].

Despite the growing recognition and positive outcomes associated with creative arts therapies, there are still challenges that need to be addressed. Limited access to these therapies, insufficient funding, and a lack of standardized training and certification are some of the barriers that need to be overcome. Besides, each therapy modality (e.g. music therapy, art therapy, etc.) advances and develops also as an individual discipline and has its own regulations according to each country and geographical region. Advocacy efforts are crucial to raise awareness, promote research, and expand the availability of creative arts therapies to a wider range of individuals and communities.

### Call for the collection series

We are excited to announce a call for submissions to our upcoming collection on mind-body interventions, specifically focusing on the integration of creative art practices in health. This collection aims to explore the transformative potential of arts interventions, creative arts therapies, or combining mind-body approaches with various art forms, highlighting the therapeutic and health-promoting benefits of this multidimensional approach.

This collection welcomes clinical studies, pilot interventions, practice reports, case studies, perspective articles, or theoretical articles that focus on broad aspects of arts in health, art interventions as therapy, and creative arts therapies including but not limited to music, dance, visual art, drama, and writing. We are also interested in studies that explore the integration of mind-body approaches, such as mindfulness, yoga, or meditation, with creative art practices to enhance therapeutic outcomes and overall well-being. Additionally, we encourage authors to submit articles investigating the underlying mechanisms and neurobiological processes involved in arts interventions in health contexts. Studies exploring the neurocognitive aspects of art making, the impact of different art materials and treatment modalities on neuro-physiological parameters, and the physiological changes associated with art interventions are of great interest. We encourage submissions from diverse disciplines and fields of research, including psychology, psychotherapy, creative arts therapies, or the health sciences, among others .

By compiling a collection of cutting-edge research and innovative approaches, we aim to contribute to the growing body of knowledge on creative arts therapy and mind-body interventions and their applications in health. We look forward to receiving your submissions and contributing to the expanding knowledge base on the integration of mind-body interventions and creative art practices. Together, we can further explore the potential of this interdisciplinary approach to promote holistic well-being, emotional healing, and personal growth. We look forward to receiving your submissions and contributing to this important field of research.

### About the collection series

This collection is part of the “Mind-body Interventions” collection series by *BMC Complementary Medicine and Therapies*. Other collections in the series include “Mind-body Interventions: Mindful Movement Practices” and “Mind-body Interventions: Mindfulness and Meditation.“ By contributing to this collection, you will be part of a growing body of knowledge aimed at advancing the understanding and application of mind-body interventions in the context of health and well-being. Submissions to this collection should follow the guidelines provided by *BMC Complementary Medicine and Therapies*. Manuscripts will undergo a peer-review process to ensure the highest scientific quality and relevance to the theme of the collection.

## Data Availability

N/A.
